# Norovirus and rotavirus in children hospitalised with diarrhoea after rotavirus vaccine introduction in Burkina Faso

**DOI:** 10.1017/S0950268820002320

**Published:** 2020-10-01

**Authors:** Y. Rönnelid, I. J. O. Bonkoungou, N. Ouedraogo, N. Barro, L. Svensson, J. Nordgren

**Affiliations:** 1 Division of Molecular Virology, Department of Biomedical and Clinical Sciences, Linköping University, Linköping, Sweden; 2 University Joseph KI-ZERBO, Ouagadougou, Burkina Faso; 3 University of Dedougou, Dedougou, Burkina Faso; 4 Division of Infectious Diseases, Department of Medicine, Karolinska Institute, Stockholm, Sweden

**Keywords:** Burkina Faso, molecular epidemiology, norovirus, prevalence, rotavirus

## Abstract

Several studies report norovirus as the new leading cause of severe gastroenteritis in children after the global introduction of rotavirus vaccines. Burkina Faso introduced general rotavirus vaccination with the oral pentavalent vaccine RotaTeq in November 2013 and quickly reached a vaccine coverage of >90%. This study describes detection rates, clinical profiles and the molecular epidemiology of norovirus and rotavirus infections in 146 children aged <5 years with severe acute gastroenteritis in Ouagadougou, consecutively enrolled from a hospital between January 2015 and December 2015. Virus detection was performed with an antigen test or real-time polymerase chain reaction (PCR) and genotyping was performed by nucleotide sequencing or multiplex PCR. Rotavirus was found in 14% and norovirus in 20% of faecal samples. Norovirus infection was significantly more associated with severe dehydration compared to rotavirus (*P* < 0.001). Among genotyped norovirus samples 48% (12/25) belonged to GII.4 which caused significantly more diarrhoeal episodes than non-GII.4 genotypes (*P* = 0.01). The most common rotavirus genotypes were G2P[4] (30%), G12P[6] (25%) and G12P[8] (20%). Fifty percent of the rotavirus positive children were infected with fully or partly heterotypic strains. In conclusion, this study found a higher proportion of norovirus causing more severe symptoms in children with diarrhoea in Burkina Faso after the introduction of rotavirus vaccination.

## Introduction

Infectious gastroenteritis is an important cause of childhood morbidity and mortality worldwide, especially in low- and middle-income countries. Rotavirus group A (hereafter called rotavirus) and norovirus are the most common viral causes of childhood diarrhoea and are every year responsible for about 300 000 to 500 000 deaths in children <5 years of age due to dehydration and electrolyte imbalances [1, 2]. Infants are generally more vulnerable than older children.

Rotavirus is divided into different G- and P-types depending on the genes encoding VP7 and VP4, two proteins in the outer shell that elicit neutralising antibodies. Since rotavirus carries a segmented genome where different segments code for different proteins, reassortment between viral strains can occur during coinfection. Today there are two internationally licensed, oral, live attenuated rotavirus vaccines available on the global market. A monovalent vaccine based on the human G1P[8] genotype (Rotarix, GlaxoSmithKline) and a pentavalent human-bovine reassortant vaccine based on G1-4P[8] (RotaTeq, Merck). The World health Organization (WHO) recommends countries with a high rotavirus disease burden to include rotavirus vaccination in their national child immunisation programmes [3]. Norovirus are ssRNA viruses that are divided into genotypes based on the sequence of the gene encoding the major capsid protein, VP1 or a dual classification system that also uses the polymerase gene and enables tracing of recombinant strains [4]. Despite a high-genetic diversity among noroviruses, the genotype GII.4 is predominant at a global level and is responsible for the vast majority of norovirus outbreaks [5]. Since 1995 eight different GII.4 variants have emerged with some years interval, where new pandemic variants have replaced the previously circulating variant. Since 2012 the predominant variant is GII.4 Sydney [4]. No norovirus vaccines or antivirals are available.

Burkina Faso is a West African low-income country with a population of approximately 20 000 000 and an annual birth cohort of about 750 000 [6]. Burkina Faso introduced rotavirus vaccination with RotaTeq in their routine immunisation programme in October 2013, with doses given at 2, 3 and 4 months, and quickly reached a coverage of >90% [7]. Before introduction, rotavirus was the leading cause of diarrhoeal-associated hospitalisations, making up roughly one-third of admissions, with a higher incidence during the cold dry period (December–April) [8].

After introduction of vaccination the proportion of rotavirus-associated hospitalisations has declined in Burkina Faso and several other sub-Saharan countries, even though the protective effect of the vaccine has been lower than in high- and middle-income countries [9]. The reason for the lower protective effect in low-income settings, and especially in sub-Saharan Africa, is not fully recognised but early infections, malnutrition and circulation of heterotypic rotavirus strains different from the G and P-types of the vaccines have been proposed [10]. In recent years the observation that human histo-blood group antigens affect susceptibility to rotavirus infection depending on rotavirus P-types offers an explanation for the lower vaccine protective effect in Burkina Faso and many other sub-Saharan countries [11]. After the global introduction of rotavirus vaccination, several studies in different countries have shown norovirus as the leading cause of gastroenteritis in children, but few studies have yet investigated this in sub-Saharan Africa [12–14].

The aim of this study was to investigate the detection rates, clinical manifestations and genetic diversity of viral gastroenteritis after introduction of rotavirus vaccine in Burkina Faso. Together with data from pre-vaccination years [15], this could provide useful information about the impact of rotavirus vaccination at a population level.

## Materials and methods

### Study design

This study was part of a larger vaccine impact and surveillance study conducted by the National Public Health Laboratory in Burkina Faso with support from WHO, Centers for Disease Control and Prevention (CDC) foundation and Gavi [16]. The study received ethical permission from the Burkina Faso Ministry of Health in the context of assessing the impact of the introduction of rotavirus vaccination in the country in 2013, according to national regulations on public health surveillance. All samples used in this study had been anonymised and made untraceable before storage. Verbal informed consent was acquired from parents or legal guardians prior to enrolment. The faecal samples used were from January to December 2015 from children aged <5 years who came to the paediatric emergency department or paediatric wards at ‘Hôpital du District de Bogodogo’ in Ouagadougou, the capital of Burkina Faso, with acute severe gastroenteritis. Acute gastroenteritis was defined as ≥3 looser-than-normal stools in a 24 h period presenting with or without vomiting and occurring for ≤7 days before arrival [17]. Severe gastroenteritis was defined by the need of hospital admission and/or intravenous fluid therapy. Hôpital du district Bogodogo is one of the four secondary health care centres in Ouagadougou and its paediatric ward has a capacity of 30 beds and admits over 2260 children each year coming from Ouagadougou city and surroundings. Children with diarrhoea who were identified >48 h after admission were not enrolled to reduce the risk of including hospital acquired infections.

### Data collection

Collection of vaccination history and clinical, demographic and socio-economical data was carried out as described in the previous vaccine evaluation study [16]. Dehydration level was determined as ‘no dehydration’, ‘moderate dehydration’, ‘severe dehydration’ or ‘shock’ based on clinical assessment of general condition, presence of sunken eyes, level of thirst and skin turgor.

### Rotavirus antigen detection

Stool samples were collected within 48 h of admission to hospital and stored at 2–8 °C (if tested within 1 week) or at −20 °C (if tested more than 1 week later). The samples were processed and tested using a commercial enzyme immunoassay (ProSpectT, Oxoid, Cambridge, UK) and afterwards stored at −70 °C at the National Public Health Laboratory in Ouagadougou, Burkina Faso.

### Rotavirus and norovirus RNA extraction and reverse transcription

Viral RNA was extracted from 140 μl of 10% stool suspensions using a QIAamp Viral RNA Mini Kit (Qiagen, Hilden, Germany) according to the manufacturer's instructions. Reverse transcription was carried out as described previously [18].

### Sequencing of rotavirus VP4 and VP7 genes and G and P genotyping by multiplex polymerase chain reaction (PCR)

PCR amplification of partial VP4 and VP7 genes was performed on samples positive for rotavirus antigen. Using primers (VP7-F, VP7-R, Con-2 and Con-3) [19] and thermocycler conditions described previously [20], 881 and 876 bp fragments were amplified from the VP7 and VP4 genes respectively. Nucleotide sequencing based on BigDye chemistry was performed by Macrogen (Amsterdam, The Netherlands) using the primers mentioned above.

Rotavirus G and P genotyping was performed on samples that could not be sequenced, by using semi-nested, type-specific multiplex PCR described previously [19], together with additional primers [15, 21]. The combination of primers were able to detect nine G-types (G1, G2, G3, G4, G6, G8, G9, G10 and G12) and six P-types (P[4], P[6], P[8], P[9], P[10] and P[11]). The rotavirus G and P genotypes were determined by their specific size on 2% agarose gel stained with SYBR safe dye (Invitrogen, Eugene, Oregon, USA) when visualised under UV light.

### Norovirus detection and geno-grouping by real-time PCR

Norovirus was detected using a modified version of a duplex TaqMan real-time PCR [22] with genogroup I and genogroup II specific probes described earlier [23]. The primers used were NVG1f1b, NVG1rlux (without hairpin), NVG2flux1 (without hairpin) [22] and COG2R [23]. Genogroup I specific probe used was 5′-[6FAM]AGATYGCGRTCYCCTGTCCA[BHQ1] and genogroup II specific probe was 5′-[HEX]TGGGAGGGCGATCGCAATCT[BHQ1] [23].

For each reaction 2 μl of cDNA was added to a reaction mixture consisting of 10 μl iTaq Universal probes Supermix 2× (Bio-Rad, Solna, Sweden), 0.8 μl of forward primer (10 pmol/μl), 0.8 μl of reverse primer (10 pmol/μl), 0.4 μl of probe (10 pmol/μl) and 4 μl of RNase-free water to make a total volume of 20 μl. The real-time PCR was performed on a CFX96 (Bio-Rad) with the following cycling conditions: initial denaturation for 10 min at 95 °C followed by 45 cycles of 95 °C for 15 s and 60 °C for 1 min. Samples were run in duplicate together with positive and negative controls. Samples with cycle to threshold (Ct-value) ≤40 were considered positive.

### Sequencing of the norovirus N-terminal and shell region

A 381 base pair fragment of the N-terminal and shell region of the capsid gene of norovirus was amplified using genogroup I specific primers NVGIF1b [22] and G1SKR [24]. A total of 3 μl of cDNA and 0.75 μl of forward and reverse primers (10 pmol/μl) and RNase-free water were added to one Illustra PuReTaq Ready-To-Go PCR Bead (GE Healthcare, Uppsala, Sweden) to obtain a final volume of 25 μl. To amplify a corresponding fragment of the NS region of genogroup II, a semi-nested PCR is developed as follows: first round: a 378 base pair fragment was amplified using forward primer QNIF2 [25] and reverse primer G2SKR [24] and second round: a 344 base pair fragment was amplified using forward primer G2SKF [24] and reverse primer G2SKR. For each reaction the same proportions of primers and PuReTaq Ready-To-Go PCR Bead (GE Healthcare) were used as described for genogroup I with 3 μl of cDNA added in the first round and 1 μl of PCR product added in the second round.

The PCRs for genogroups I and II were performed under the following conditions: 94 °C for 4 min followed by 40 cycles of 94 °C for 30 s, 50 °C for 30 s and 72 °C for 1 min, with a final extension step at 72 °C for 7 min. The amplicons were visualised on 2% agarose gel stained with SYBR safe dye (Invitrogen) and subsequently sent to Macrogen for nucleotide sequencing. The norovirus strains were later genotyped using an online norovirus-genotyping tool [26] and/or with phylogenetic analysis described below.

### Phylogenetic analyses

Multiple sequence alignment of the partial VP4 and VP7 genes of rotavirus and the partial N-terminal and shell region of norovirus was performed using the ClustaIW algorithm in BioEdit software version 7.2.5. Suitable evolutionary model was determined in MEGA7 software and the same programme was subsequently used to construct phylogenetic trees using the maximum likelihood method with Tamura 3-parameter and invariant sites for rotavirus and Kimura 2-parameters and gamma distributed rate heterogeneity for norovirus. The reliability of the topologies of the phylogenetic trees was inferred by bootstrapping with 100 pseudo-replicates. GenBank accession numbers for norovirus are MN744428 (genogroup IX) and MN744436–MN744456 (genogroup II), and for rotavirus are MN746335–MN746349 (VP4) and MN758622–MN758636 (VP7).

### Statistical analysis

Categorical variables were analysed using the Fisher's exact test if the two variables had two categories each, or chi-square test if the variables had more than two categories. As we had a limited sample size, some categorical variables such as children's dehydration level were condensed into two groups (no and moderate dehydration *vs.* severe dehydration and shock) that were considered more clinically relevant. Quantitative variables are presented as mean with standard deviations, and independent samples *t*-test was used to test differences between two groups if the variables followed normal distribution as determined by the Shapiro–Wilk's test. Otherwise, the variables were presented as median, and Mann–Whitney *U* test was used to test differences between two or more groups. Statistical analysis was performed with a two-tailed significance using the software SPSS, version 26. *P* values <0.05 were considered significant.

## Results

### Norovirus was more common than rotavirus in children hospitalised with gastroenteritis

A population of 151 children with severe acute gastroenteritis was included in the study. Stool samples from five of the subjects could not be retrieved, leaving 146 participants in the final analysis. Rotavirus was found in 14% (20/146) and norovirus in 20% (29/146) of the faecal samples, with no co-infections found. Norovirus was more common in the age group 7–12 months, where it was found in 27% (17/63) of the samples and was not found at all in the age group over 24 months while rotavirus was found in all age groups and varied between minimum 10% (6/63) in the age group 7–12 months and maximum 18% (4/22) in the age group 0–6 months (Table 1).

**Table 1.** Socio-demographic characteristics of children <5 years with acute severe gastroenteritis infected with norovirus or rotavirus

Socio-demographic characteristics	Total *n* = 146 (%)	Norovirus positive *n* = 29 (%)	Rotavirus positive *n* = 20 (%)
*Age range (in months)*			
≤6	22 (15)	5 (23)	4 (18)
7 to 12	63 (43)	17 (27)	6 (10)
13 to 24	43 (29)	7 (16)	7 (16)
>24	18 (12)	0 (0)	3 (17)
*Sex*			
Female	70 (48)	15 (21)	10 (14)
Male	76 (52)	14 (18)	10 (13)
*Mothers education*			
None or primary school	96 (66)	14 (15)	15 (16)
Secondary school or university	50 (34)	15(30)	5 (10)

All of the rotavirus positive samples were detected during the cold dry season and the following months (between January and May) with a peak detection rate in January when rotavirus was found in 57% (8/14) of screened samples. Norovirus was detected more evenly throughout the year with a peak in December when it was found in 83% (5/6) of screened samples.

The norovirus positive children had the highest proportion of mothers with secondary school education or higher (Table 1), but other socio-economic data like access to electricity or the source of drinking water did not differ between groups. Among mothers with secondary school education or higher, 68% (34/50) had children who were immunised with a full three-dose regimen of rotavirus vaccine compared with 55% (53/96) of the mothers with primary school education or lower.

### Norovirus caused more severe symptoms than rotavirus

Of all included children, 70% (102/146) had received at least one dose of rotavirus vaccination, with 70% (14/20) of rotavirus positive children and 85% (25/29) of norovirus positive children receiving at least one dose of rotavirus vaccine (Table 2). Norovirus positive children had also completed the full three-dose vaccination regimen in a higher extent than rotavirus positive children with 72% (21/29) of norovirus positive and 55% (11/20) of rotavirus positive having completed the full vaccination regimen. In the rotavirus positive group, 30% (6/20) had a concomitant malaria infection as compared with 17% (5/29) in the norovirus positive group and 20% (19/97) in the group with diarrhoea of other reasons (Table 2). Norovirus and rotavirus positive and the group with other aetiologies were associated with fever (≥38 °C), in 86%, 90% and 82% of cases, respectively. Children positive for norovirus were significantly more likely to be severely dehydrated (severe dehydration or shock) compared with rotavirus positive children where no child was severely dehydrated (Table 2). In addition, norovirus positive children had more frequent vomiting than rotavirus positive children with a median of 4 and 3 episodes per day, respectively. Diarrhoea was also more common in norovirus positive children who had a median of 5 diarrhoeal episodes per day compared with rotavirus positive children with a median of 4 diarrhoeal episodes per day (Table 2).

**Table 2.** Clinical profiles of norovirus and rotavirus children <5 years with acute severe gastroenteritis

Clinical features	Norovirus positive *n* = 29 (%)	Rotavirus positive *n* = 20 (%)	Other *n* = 97 (%)	*P*-value^a^
Fever ≥ 38 °C	25 (86)	18 (90)	80 (82)	1.0^b^
Malaria	5 (17)	6 (30)	19 (20)	0.32^b^
Rotavirus vaccination				
No vaccination	4 (14)	6 (30)	34 (35)	0,16^c^
Partial vaccination^d^	4 (14)	3 (15)	8 (8)	
Full vaccination^e^	21 (72)	11 (55)	55 (57)	
*Dehydration level*				
No-moderate	16 (55)	20 (100)	59 (61)	0.0003^b^
Severe-shock	13 (45)	0 (0)	38 (39)	
Vomiting	21 (72)	15 (75)	52 (54)	1.0^b^
Vomiting episodes (median)	4	3	3	0.56^f^
Diarrhoea episodes (median)	5	4	4	0.15^f^

a Comparing norovirus and rotavirus positive groups.

b Fisher's exact test.

c Chi-square tests.

d 1–2 doses.

e 3 doses.

f Mann–Whitney *U* test.

### High-genetic diversity of norovirus, with genotype GII.4 associated with more severe symptoms

**Fig. 1.**
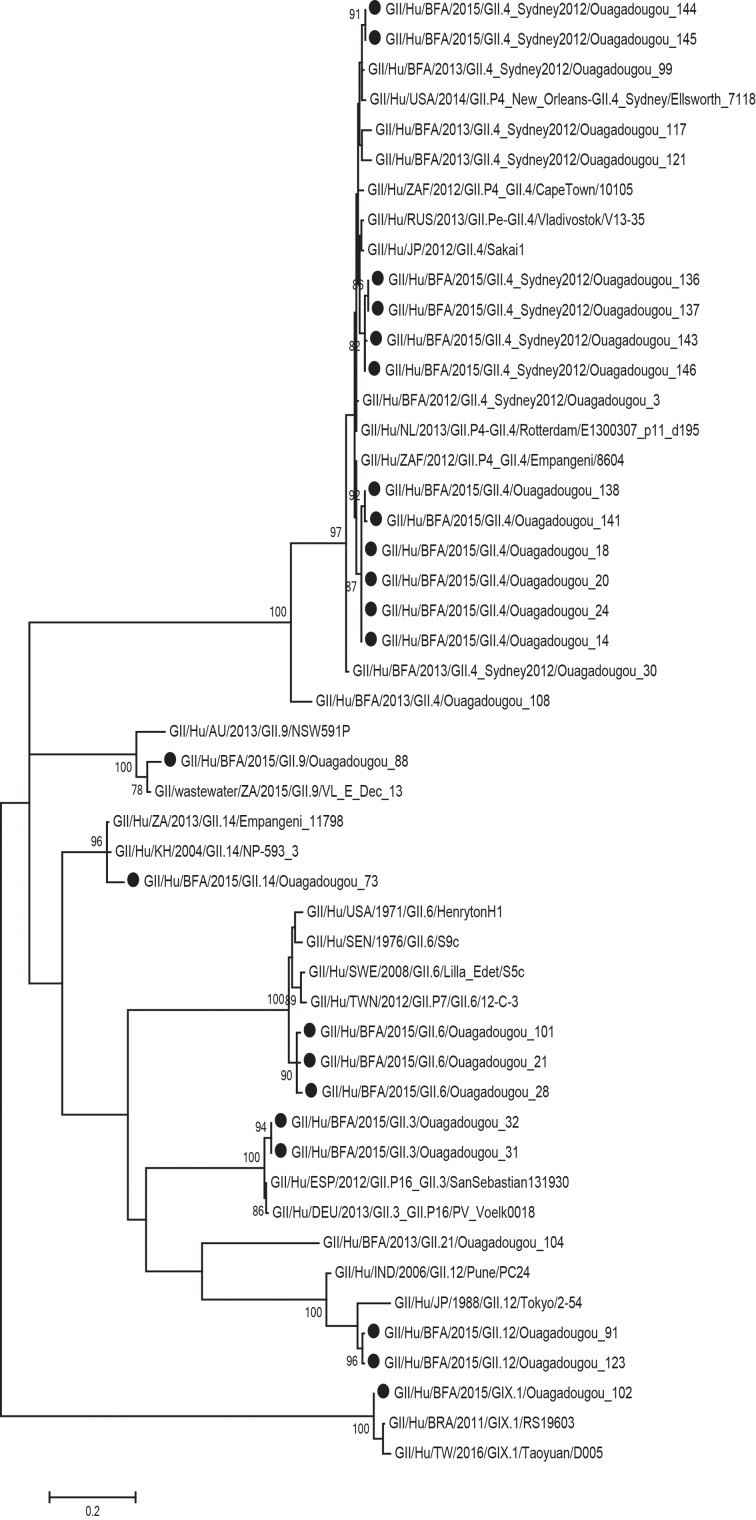
Phylogenetic analysis of the N-terminal and shell region (nt 1-272; ORF2) of norovirus genogroup II and IX strains in Burkina Faso January–December 2015. The tree contains most similar norovirus strains found on GenBank and norovirus strains from Burkina Faso 2013. The norovirus strains from this study are marked with filled circles. Scale bars represent the number of substitutions per site and bootstrap values are shown at branch nodes (values <70% are not shown).

Of the 29 norovirus positive samples 25 belonged to genogroup II, three belonged to genogroup I and one belonged to genogroup IX. Furthermore, 21 of the genogroup II samples, and the genogroup IX sample could be genotyped based on sequences of the N-terminal and shell region and all of these had a sequence length over 270 base pairs and were included in the phylogenetic analysis (Fig. 1). Of genogroup II, 57% (12/21) samples were of genotype GII.4, all the Sydney_2012 variant, three samples belonged to GII.6, two samples belonged to GII.3, two samples belonged to GII.12 and one sample each belonged to GII.9 and GII.14. Of the samples with genogroup I, two samples belonged to genotype GI.3 and one to genotype GI.5. Children with GII.4 genotype infections had significantly more diarrhoeal episodes (median 6 episodes) than children with non-GII.4 genotypes infections (median 4 episodes) (Table 3). The mean CT-value for samples with GII.4 was lower (25.6) compared to that of non-GII.4 (28.7), although the difference was not statistically significant. The age profile of children with GII.4 genotype was similar to children with non-GII.4 genotypes with all infections occurring in children under 2 years of age. Most norovirus positive children had fever regardless of genotype (Table 3).

**Table 3.** Clinical features and mean CT-values of norovirus positive children

	Non-GII.4 *n* = 13 (%)	GII.4 *n* = 12 (%)	*P*-value
*Age range (in months)*			
≤6	2 (15)	3 (25)	0.66^a^
7 to 12	7 (54)	7 (58)	
13 to 24	4 (31)	2 (17)	
>24	0	0	
Fever ≥ 38 °C	11 (85)	10 (83)	1.0^b^
*Dehydration level*			
No-moderate	7 (54)	6 (50)	1.0^b^
Severe-shock	6 (46)	6 (50)	
Vomiting	8 (61,5)	11 (91,7)	0.16^b^
Vomiting episodes (median)	4	4	0.72^c^
Diarrhoea episodes (median)	4	6	0.01^c^
Mean CT-value ± s.d.	28.7 ± 4.4	25.6 ± 4.8	0.12^d^

a Chi-square tests.

b Fisher's exact test.

c Mann–Whitney *U* test.

d Independent samples *t*-test.

### G2p[4] is the most common rotavirus genotype, followed by G12P[6] and G12P[8]

**Fig. 2.**
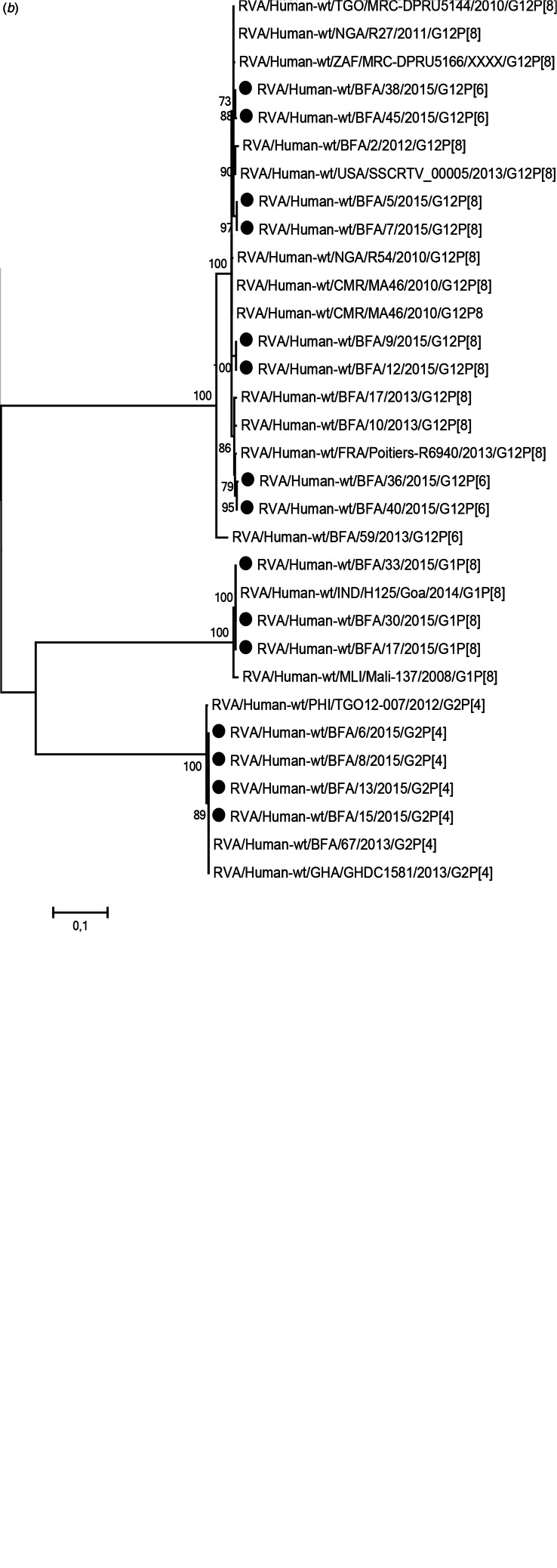
Phylogenetic analysis of sequences of rotavirus. (a) VP4 gene (nt 51-802) and (b) VP7 gene (nt 209–844). The phylogenetic tree contains most similar rotavirus strains found on GenBank and rotavirus strains from Burkina Faso 2013. Scale bars represent the number of substitutions per site and bootstrap values are shown at branch nodes (values <70% are not shown).

Of the 20 samples that were positive for rotavirus antigen, 19 samples could be successfully genotyped by either sequencing of partial VP4 and VP7 capsid genes or by multiplex PCR. The distribution of rotavirus G and P types is shown in Table 4. The G12 genotypes belonged to lineage III. The genotypes G2P[4] and G12P[8] predominated in the beginning of the year while all of the P[6] types (G12P[6] and G1P[6]) occurred in the end of the cold and dry season (March–May). The median age was 7 months for children infected with P[8], 16.5 months for infection with P[6] and 20 months for infection with P[4]. Of the nucleotide sequences of the rotavirus capsid genes VP4 and VP7, 15 sequences from each gene had sufficient length and quality and were included in the phylogenetic analysis (Figs 2a and 2b). The VP7 genes of the G12P[8] isolates were grouped into two different phylogenetic clusters exhibiting 1.7% nucleotide difference. The VP4 genes of the same samples (isolate 5, 7, 9 and 12) were similarly grouped into two clusters. The VP7 genes of the G12P[6] isolates was clustering in two different groups with two isolates in each group differing 1.6–1.9% in nucleotide sequence, while the VP4 gene of the same isolates grouped together in one cluster of three isolates and one branch with only one isolate differing 4.3–4.5% in nucleotide sequence. The VP7 genes of the G2P[4] isolates were identical to each other and to strains circulating in Burkina Faso and Ghana in 2013 (Figs 2a and 2b).

**Table 4.** Distribution of G and P types of rotavirus group A strains detected among children with severe gastroenteritis in Ouagadougou January 2015–December 2015

Rotavirus genotype	No. (%) of strains				
	P[4]	P[6]	P[8]	P[nt]	Total
G1	0	1	3	0	4 (20)
G2	6	0	0	0	6 (30)
G12	0	5	4	0	9 (45)
Gnt	0	0	0	1	1 (5)
Total	6 (30)	6 (30)	7 (35)	1 (5)	20 (100)

Nt, not determined.

## Discussion

This study found that norovirus was more commonly detected and associated with more severe symptoms compared to rotavirus in children in Burkina Faso after rotavirus vaccine introduction. The high norovirus prevalence corresponds well with other studies in high- and middle-income countries where norovirus has become the most common aetiology in severe childhood diarrhoea [12–14]. Other studies from Malawi and Tanzania have shown lower prevalence of norovirus compared to this study, but used other detection methods that might not be directly comparable [27, 28].

Most of the rotavirus positive children (55%) in this study were symptomatically infected despite having received a full three-dose regimen of rotavirus vaccine, indicating a less than optimal protective effect of the vaccine. This study was a part of a larger 3 year multicentre study which showed an overall vaccine effectiveness of 35% in children aged 6 months to 5 years and a higher effectiveness, 58%, among younger children aged 6–11 months [16]. These findings are similar to the reported vaccine effectiveness in Ghana, Zambia and Botswana [16]. In this study the highest proportion of norovirus positive children was found in the age group 7–12 months which is a similar age distribution as was found in the pre-vaccination year 2013 [15]. When comparing the findings before and after vaccine introduction it appears that the age distribution for rotavirus has shifted with a majority (67%) of symptomatic children aged 6–12 months being positive for rotavirus in 2013, declining to 10% in 2015 [15]. This apparent decline of symptomatic rotavirus infection among young children corresponds well with the relatively high vaccine effectiveness observed for the same age group [16].

This study found norovirus positive children with symptomatic infection to be significantly more dehydrated than the rotavirus positive children. Furthermore, norovirus genotype GII.4 had significantly more diarrhoeal episodes than non-GII.4 genotypes. This finding supports the suggestion that GII.4 genotypes cause more severe gastroenteritis than other norovirus genotypes. Earlier studies have found that GII.4 genotypes are associated with longer duration of diarrhoea and vomiting [29], being more commonly observed in hospitalised patients than in community settings [12], causing infection with a higher viral load and more often causing dehydration that needs intravenous fluid therapy [30].

In this study a norovirus of the rare genotype GIX.I was detected, until recently classified as GII.15 [4]. This is the first GIX.I strain that has been detected in sub-Saharan Africa as reported to the NCBI GenBank or its connected databases.

In humans histo-blood group antigens affect susceptibility to norovirus infections, with some genotypes like GII.4 and GI.1 infecting almost exclusively secretors, and other genotypes such as GII.2 or GI.3 infecting persons independent of secretor status [31]. There are norovirus vaccines in development, where two candidates have progressed to clinical trials in people. A bivalent vaccine (GI.1 and GII.4) based on virus like particles that is administered parenterally has been shown to be safe, immunogenic and to reduce diarrhoea and vomiting in a GII.4 challenge study in secretor positive volunteers [32]. A parenteral administration like this vaccine probably circumvents the problem of including only secretor-dependent genotypes in a vaccine that should also elicit protective immunity in secretor negative individuals. The recent and much-needed development of human intestinal enteroids, an *in vitro* model that supports norovirus cultivation, opens up the future development of live, attenuated norovirus vaccines [33]. A future live oral vaccine should preferably be based on the globally most important GII.4 genotype in combination with a secretor independent genotype to avoid a failed vaccine take and no or inadequate protective immunity in secretor negative individuals.

Of the rotavirus G-types 45% belonged to G12, 25% being G12P[6] and 20% G12P[8], a G-type that emerged globally around 2012 [15, 34, 35] and was rare when today's vaccines were formulated and thus not included in vaccine formulations. Including strains with G1P[6], 50% of the rotavirus positive children in this study were infected with fully or partly heterotypic strains. A study of children with diarrhoea in Nicaragua, after general rotavirus vaccination with an immunisation rate of >90%, found that during 2012–2013 a majority of symptomatically infected children had the newly emerging G12 G-type. Of the G12 infected children 75% had a high anti-rotavirus IgA titre in faecal samples indicating that they were either vaccinated or had been previously infected with wild type rotavirus strains, but were not protected from symptomatic G12 infection [34]. A systematic review and meta-analysis from 2014 did not find any significant difference in effectiveness between homotypic, fully or partly heterotypic strains for either RotaTeq or Rotarix [36], but included only one study with G12 and only two studies with few subjects being infected with P[6] types. Also, early clinical trials suggesting a similar effect to homo and heterotypic strains [37] were conducted during a time when the five strain combinations G1P[8], G2P[4], G3P[8], G4P[8] and G9P[8] were accounting for over 75% of rotavirus infections in children, before the global increase of G12 and G9 [38]. It is thus not possible to exclude that circulation of heterotypic strains affect the vaccine effect and need to be studied further.

In recent years, another plausible explanation of low vaccine effect in populations with a high proportion of non-secretor and Lewis negative individuals has been hypothesised. Histo-blood group antigens has been shown to affect susceptibility to rotavirus infection in a P-type dependent manner, with P[8] mainly infecting Lewis and secretor positive children and P[6] infecting mainly Lewis negative children [11]. Studies have shown a lower vaccine take for non-secretors [39, 40] and it is thus possible that vaccinated Lewis and secretor negative children can be subsequently symptomatically infected with P[6] rotaviruses, common in sub-Saharan Africa [11].

Interestingly, rotavirus infections with P[4] and P[8] types occurred in the beginning of the season while all infection with P[6] types occurred at the end of the rotavirus season (March–May), a pattern that has been shown earlier in Burkina Faso during several years [15, 41]. Data from this and earlier studies also suggest that the age group for infection with P[6] genotypes are changing between years. In 2010 P[6] rotaviruses infected older children [41], while in 2013 P[6] rotaviruses infected younger children [15]. In this study enrolling children during 2015, P[6] was again infecting older children (median age 16.5 months) compared to P[8] (median age 7 month). The reasons for these fluctuations in age groups between P-genotypes are not known but could be associated with herd immunity, related to P-types infecting different populations based on expression of histo-blood group antigens.

Overall, the phylogenetic analysis of rotavirus VP7 and VP4 reveals a greater diversity in 2015 than in the pre-vaccination year 2013 [15]. The finding that the G12P[6] isolate 38 was nearly identical (differing 0.2% in nucleotide sequence) to the G12P[6] isolate 45, but clustering in their own group differing over 4% in nucleotide sequence with the other G12P[6] isolates, suggests a previous reassortant event. There was a predominance of G12 of lineage III, in different P-type combinations and in four distinct phylogenetic clusters (both for VP4 and VP7), indicating introduction of G12 viruses of four different ancestral strains. This highlights the ongoing evolution and widespread transmission of this genotype in the post-vaccination era, and the need for continued surveillance.

A further noteworthy finding is that the G6 strains emerging during 2009–2010 that made up about one-third of the rotavirus strains in the pre-vaccination year 2013, were not detected in this study.

A limitation of this study is that there was no asymptomatic control group, leading to the possibility of overestimating the detection rate of rotavirus or norovirus as symptomatic infection since these viruses can also be found in asymptomatic carriers. Another limitation is the relatively small sample size. Furthermore, the enrolled children were not screened for other pathogens causing diarrhoea, so it was not possible to account for co-infections [28].

In conclusion this study found a higher proportion of norovirus causing more severe symptoms in children with diarrhoea in Burkina Faso after the introduction of rotavirus vaccination, highlighting the increased importance of norovirus in paediatric diarrhoea after rotavirus vaccination.

## Data Availability

The data that support the results are available from the corresponding author on reasonable request.
